# A strategy to increase adoption of locally-produced, ceramic cookstoves in rural Kenyan households

**DOI:** 10.1186/1471-2458-12-359

**Published:** 2012-05-16

**Authors:** Benjamin J Silk, Ibrahim Sadumah, Minal K Patel, Vincent Were, Bobbie Person, Julie Harris, Ronald Otieno, Benjamin Nygren, Jennifer Loo, Alie Eleveld, Robert E Quick, Adam L Cohen

**Affiliations:** 1 Epidemic Intelligence Service, Scientific Education and Professional Development Program Office, 1600 Clifton Road, Atlanta, GA 30333, USA; 2 Respiratory Diseases Branch, National Center for Immunization and Respiratory Diseases, 1600 Clifton Road, Mailstop C-09, Atlanta, GA 30333, USA; 3 Nyando Integrated Child Health and Education Project, P.O. Box 3323, 40100, Kisumu, Kenya; 4 Enteric Diseases Epidemiology Branch, National Center for Emerging and Zoonotic Infectious Diseases, Centers for Disease Control and Prevention, 1600 Clifton Road, Atlanta, GA, 30333, USA; 5 Safe Water and AIDS Project, P.O. Box 3323, 40100, Kisumu, Kenya

## Abstract

**Background:**

Exposure to household air pollutants released during cooking has been linked to numerous adverse health outcomes among residents of rural areas in low-income countries. Improved cookstoves are one of few available interventions, but achieving equity in cookstove access has been challenging. Therefore, innovative approaches are needed. To evaluate a project designed to motivate adoption of locally-produced, ceramic cookstoves (*upesi jiko*) in an impoverished, rural African population, we assessed the perceived benefits of the cookstoves (in monetary and time-savings terms), the rate of cookstove adoption, and the equity of adoption.

**Methods:**

The project was conducted in 60 rural Kenyan villages in 2008 and 2009. Baseline (*n* = 1250) and follow-up (*n* = 293) surveys and a stove-tracking database were analyzed.

**Results:**

At baseline, nearly all respondents used wood (95%) and firepits (99%) for cooking; 98% desired smoke reductions. Households with *upesi jiko* subsequently spent <100 Kenyan Shillings/week on firewood more often (40%) than households without *upesi jiko* (20%) (p = 0.0002). There were no significant differences in the presence of children <2 years of age in households using *upesi jiko* (48%) or three-stone stoves (49%) (p = 0.88); children 2–5 years of age were less common in households using *upesi jiko* versus three-stone stoves (46% and 69%, respectively) (p = 0.0001). Vendors installed 1,124 *upesi jiko* in 757 multi-family households in 18 months; 68% of these transactions involved incentives for vendors and purchasers. Relatively few (<10%) *upesi jiko* were installed in households of women in the youngest age quartile (<22 years) or among households in the poorest quintile.

**Conclusions:**

Our strategy of training of local vendors, appropriate incentives, and product integration effectively accelerated cookstove adoption into a large number of households. The strategy also created opportunities to reinforce health messages and promote cookstoves sales and installation. However, the project’s overall success was diminished by inequitable and incomplete adoption by households with the lowest socioeconomic status and young children present. Additional evaluations of similar strategies will be needed to determine whether our strategy can be applied equitably elsewhere, and whether reductions in fuel use, household air pollution, and the incidence of respiratory diseases will follow adoption of improved cookstoves.

## Background

Unprocessed biomass (e.g., charcoal, wood, crop waste) remains a major household fuel source for most residents of low-income countries, particularly the rural poor [1]. During cooking, inadequate ventilation and incomplete combustion through the use of rudimentary stoves or open firepits are common, resulting in acute and chronic exposures to air pollutants (particulate matter, carbon monoxide, nitrous oxides) [2,3]. Exposure to household air pollution has been linked to a range of negative health outcomes in children and adults, including pneumonia, tuberculosis, chronic obstructive pulmonary disease, lung cancer, low birth weight, and premature mortality [1,4-7]. Altogether, the burden of illness associated with household air pollution represents approximately 4% each of all deaths and disability-adjusted life-years in low-income countries [8]. This burden is greatest among women and young children who are usually present near the cooking site [9].

For decades, numerous designs of improved cookstoves have been widely introduced in Latin America, Africa, and Asia. However, an ideal improved cookstove design has not been established (and a universally-acceptable design may never exist) [10]. An ideal design would substantially reduce fuel use, household air pollution emissions, and cardiopulmonary disease incidence in children and adults as well as meet affordability and acceptability prerequisites among users. The unique challenges and mixed results of these first-generation undertakings have been summarized [11,12], but published reports of processes and mechanisms for equitable access to improved cookstoves in the developing world are still needed [13,14]. In particular, many cookstove designs require materials and labor for local production and installation, making the technology adoption process more complex than other product-based interventions that can be adopted immediately at the point of use.

To improve child survival in Western Kenya, a partnership was established in 2007 between the Safe Water and AIDS Project (SWAP) and the Nyando Integrated Child Health and Education (NICHE) project. SWAP is a Kenyan non-government organization that provides training, outreach and mobilization, and microfinance for community-based groups, including human immunodeficiency virus (HIV) support groups, that promote, sell, and distribute child survival products at the household level (e.g., *WaterGuard*^TM^ and other water treatment products, insecticide-treated bed nets, and micronutrient supplementation packets) (http://www.swapkenya.org). NICHE is sponsored by the U.S. Centers for Disease Control and Prevention and designed to evaluate, through weekly visits by trained field surveyors, the impact of integrated delivery of these products on population health. Product integration is the co-promotion, distribution, and use of several products or technologies designed to improve health by sharing programmatic resources and targeting the same individuals or families with multiple interventions. Integrating cleaner-burning, efficient cookstoves and household water treatment products could increase child survival by improving air and water quality to reduce rates of pneumonia [15,16] and diarrhea [17], respectively. The use of improved cookstoves is also appealing because it may translate to savings in time and money used for gathering or purchasing fuel.

Accordingly, we utilized the SWAP and NICHE partnership to evaluate a strategy of mobilizing local vendors, using pricing incentives and promotional offers, and integrating locally-produced, ceramic cookstoves with household water treatment interventions in a project designed to increase the purchase and installation (i.e., adoption) of cookstoves in Nyanza Province from 2008–2009. The three objectives of the evaluation were to assess the perceived monetary and time savings benefits of the cookstoves (i.e., fuel expenditures and time saved gathering fuel and cooking), monitor the rate of adoption of the cookstoves, and assess the equity of cookstove adoption by comparing select demographic and socioeconomic characteristics among households that did and did not purchase and install the cookstoves (e.g., presence of young children).

## Methods

### Project setting and design

Nyanza Province is located in western Kenya near the city of Kisumu. The province is typical of rural areas of Africa where women and children are exposed to household air pollution. In Nyanza, rates of acute respiratory infections, diarrheal illnesses, malnutrition, and infant and child mortality are among the highest in Kenya, and malaria transmission is endemic [18,19]. Access to health interventions in Nyanza is inadequate due to poverty and limited transportation and communication infrastructure. At least half of households rely on superficial sources of drinking water that require treatment [18]. In this polygamous society of Luo ethnicity, families live in multigenerational compounds called *dalas*, which consist of a single main house surrounded by 1 to 3 additional households. Villages usually include 60 to 110 households.

In January 2007, NICHE participant households were enrolled using a two-stage, cluster-sampling procedure. In the first sampling stage, 60 villages from 17 sublocations in the Nyando Division of the Nyanza Province were selected. Villages were randomly selected with probabilities proportional to size, based on a 1999 census of Nyando. In the second stage, 25 households with children 6–35 months of age were randomly selected per village to complete NICHE enrollment. The resulting study population consisted of members of approximately 1,500 randomly-chosen households in 60 NICHE villages located in Nyando.

Institutional review boards at the Kenya Medical Research Institute and the U.S. Centers for Disease Control and Prevention reviewed and approved the protocol. We obtained verbal informed consent from all participating households.

### Cookstove and water quality interventions

**Figure 1:**
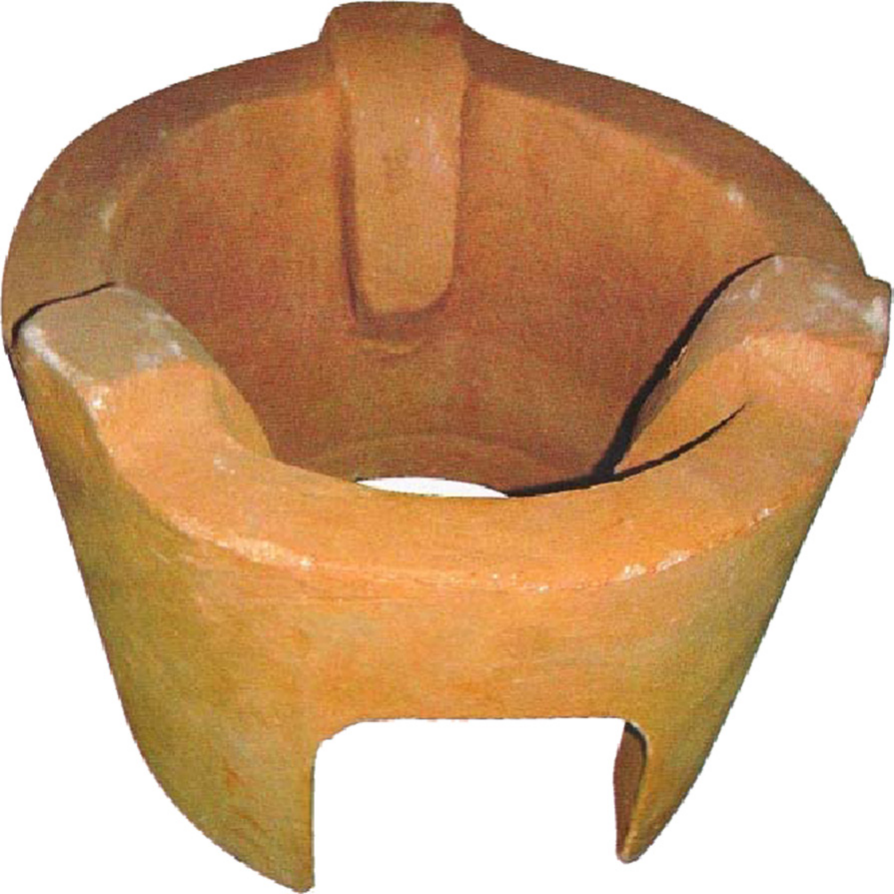
***Upesi jiko *(ceramic-lined cookstoves), Nyanza Province, Kenya **^**a **^**.**^a^ The specific dimensions of the stove are 26 centimeters diameter for the internal bottom, 30 centimeters for the internal diameter, 15 centimeters curved for the door size, 1 centimeter for the height of the pot rest, and 18 centimeters for the height of the firebox.

The cookstove technology promoted and distributed by SWAP was *upesi jiko* (Swahili for “quick stove”). The functional unit of the *upesi jiko* is a simple ceramic liner (i.e., no other parts are required). Using clay found in nearby riverbanks, these units were produced locally by skilled laborers in the Keyo and Masogo pottery groups, which are located in the cities of Kisumu and Ahero, respectively. Pottery skills are developed similarly to a trade organization with informal apprentices, journeymen, and masters. The ceramic liners (Figure1) are installed into a simple, earthen base that is constructed semi-permanently within a kitchen. The ceramic liner dimensions are guided by the Kenya Bureau of Standards (KS 1814:2005) (http://www.kebs.org), which aims to ensure that the correct shape and size are retained so that energy-saving efficiency is maintained in the design. Practical Action, a non-governmental organization that has promoted improved cookstoves for low-income countries (including Kenya), calculated that yearly savings of 7,400 Kenyan shillings (KSh) (~$100.00 USD) could be obtained by improving the efficiency of fuel use with *upesi jiko*[20]. However, the health impact of *upesi jiko* or similar cookstoves in rural Africa has not been fully established [16,21]. The relatively low cost of 150 KSh, or ~ $2.00 U.S. dollars (USD), is a primary advantage of the liners. However, additional material and labor costs for the installation of the liner into a base typically add $1.50 to $3.00 USD to the total cost. Until August 2009, liner purchase and installation were separate transactions.

*WaterGuard*^TM^, a sodium hypochlorite solution that costs $0.27 USD to treat 1,000 liters, was the primary water treatment technology that was integrated with the *upesi jiko* project. Water treatment with sodium hypochlorite solution has been associated with a reduced risk of diarrhea in Kenya, Uganda, and Zambia [22-24]. The product *WaterGuard*^TM^ is produced locally, marketed by Population Services International, and has been promoted and sold throughout Nayanza Province since 2003. A second chlorine-based water treatment technology promoted was sodium dichoro-isocyanurate, which is prepared in the form of effervescent tablets designed to treat 20 liters. The tablets were given the brand name *Aquatabs™* and sold commercially at a price of approximately $0.02 USD per tablet.

### Development and integration of cookstove market

**Figure 2:**
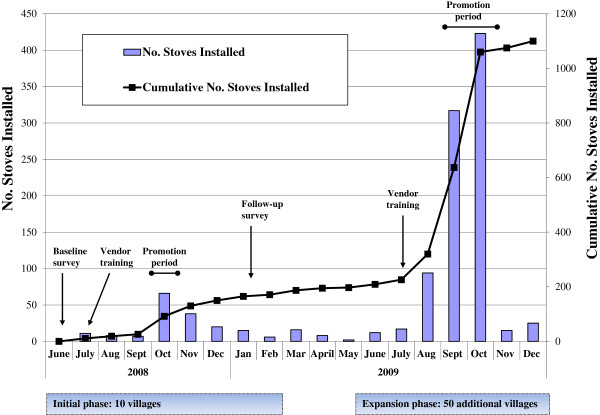
**No. of *upesi jiko *installed in all 60 villages, Nyanza Province, Kenya, July, 2008–December, 2009**^**a**^**.**^a^*Upesi jiko* are ceramic-lined cookstoves that are produced locally. Sales dates were not available for 9 transactions.

To develop and integrate the market for *upesi jiko*, we implemented and evaluated a two-stage project from June 2008 through December 2009 (Figure2). In the pilot stage (June 2008–February 2009), we engaged SWAP-affiliated members of HIV self-help groups who were already promoting and selling other child survival products. A random sample of 10 NICHE project villages was selected for implementation of the air and water quality interventions in the pilot stage. In June and July 2008, existing SWAP groups in each village identified 34 SWAP vendors to be trained immediately by NICHE staff in developing a small business to promote, sell, and install the *upesi jiko* in addition to other products. Apart from their good standing in the community and initiative, no minimum criteria were required of SWAP vendors. To demonstrate correct installation, at least one *upesi jiko* was installed in a household in each village; these demonstrations resulted in 20 trained installers (many of whom were also vendors). SWAP vendors were educated by NICHE staff about prevention of respiratory and diarrheal diseases; vendors were also trained on the principles of water treatment as well as the proper use of *WaterGuard*^TM^ and *Aquatabs™* (used more and less frequently in Nayanza Province, respectively). Following the trainings, vendors received initial stocks of four *upesi jiko* free of charge to raise capital to initiate their businesses. Subsequently, *upesi jiko* were typically purchased for 150–200 KSh by the vendors. Immediately following the completion of pilot trainings, October 2008 was intentionally designated “Stove Promotion Month” to encourage SWAP vendors and their potential customers in all 10 villages to act upon time-limited, price incentives and promotional offers. During this period, the wholesale price of *upesi jiko* was reduced from 200 to 150 KSh and SWAP vendors were also eligible for a T-shirt when they had installed their first 25 *upesi jiko*. In addition, purchasers of *upesi jiko* received a voucher with each purchase, which could be redeemed for a water treatment product (either one bottle of *WaterGuard*^TM^ or 10 *Aquatab*™ tablets). The promotion was subsequently extended to include November and December of 2008.

Additional program funding marked the beginning of an expansion stage (June–December 2009). During the expansion stage, we trained another 126 SWAP vendors in the remaining 50 NICHE project villages on promotion, sales, and installation of *upesi jiko* (i.e., 160 total vendors). From August through October 2009, SWAP vendors were offered one T-shirt for every three *upesi jiko* installed within one week; *upesi jiko* wholesale prices were also reduced to 150 KSh. *Upesi jiko* purchasers were offered a discount of 100 KSh from the usual price through a subsidy that was awarded to the SWAP vendors upon installation of each *upesi jiko*. *Upesi jiko* sales and installations were combined into a single transaction in August 2009 to decrease the time interval between these activities. In the expansion stage, *upesi jiko* were promoted, sold, and installed in all 60 villages.

### Monitoring and evaluation

To assess the strategy for increasing adoption of *upesi jiko*, we prospectively followed the cohort of households to monitor cookstove adoption and the integration of water treatment practices.

#### Baseline and follow-up surveys

NICHE field surveyors conducted cross-sectional surveys of cooking locations, fuel costs, time spent gathering fuel and cooking, and exposures to smoke at baseline and during follow-up. A baseline survey of cookstove use was performed in June 2008 to determine whether a need for improved cookstoves existed within the NICHE participant villages. During home visits, NICHE field surveyors used a standardized questionnaire to interview women who were primarily responsible for cooking and childcare. Respondents from 1,500 households in the 60 villages that would have access to *upesi jiko* during the pilot or expansion stages were eligible for enrollment in the baseline survey.

A follow-up survey was implemented in January and February of 2009 as an evaluation of the completion of the pilot stage. The questionnaire was similar in content to the baseline instrument, but limited to the 10 villages that were included in the pilot stage. All households participating in NICHE and any household (NICHE and non-NICHE) that purchased an *upesi jiko* was invited to participate in the survey. Survey data from households using an *upesi jiko* were compared with households that were still using a traditional, open firepit with three surrounding stones (hereafter referred to as three-stone stove) for cooking; cooking locations, fuel costs, time spent gathering fuel and cooking, and exposures to smoke were compared to determine whether the cookstoves were perceived to be beneficial.

#### Cookstove transaction tracking

To monitor the rate of cookstove adoption, stove transactions (sales and installations) were tracked by trained NICHE field surveyors during bi-monthly household visits from July 2008 through December 2009. Two data managers were trained on maintenance of a *Microsoft Access* database for the stove transactions (Microsoft Corp, Redmond, WA); data quality was sustained through quarterly assessments.

### Data analysis

To assess whether there was a potential impact from the co-promotion of *upesi jiko* and household water treatment on levels of cookstove adoption, adoption of *upesi jiko* was compared among frequent, sporadic, and never-treaters of water, which were defined as having reported water treatment in the last 12 hours during at least half (≥50%) of NICHE field surveyor visits, less than half (<49%) of visits, and never, respectively. Stove installations were assessed among the subset of households that already had access to water treatment products and had participated in at least 50% of NICHE household visits between March 2008 and March 2009. This subset of more active NICHE-participant households was visited at least 9 times (i.e., 9 of 18 possible visits), which allowed for a more stable determination of actual household water treatment over time. Because self-reported water treatment may be overly reported (i.e., social desirability bias), the use of a household water treatment product was confirmed by measuring chlorine residual with an N, N diethyl-p-phenylene diamine (DPD) color comparator test kit (LaMotte Co., Maryland). Chlorine residuals were measured during each visit. Using the same cutpoints described above, similar categories of water treatment (frequently-confirmed, sporadically-confirmed, and never-confirmed treaters) were defined for chlorine residual measurements.

To assess the equity of cookstove adoption, a principal component analysis of household assets (e.g., household goods, building structure) was used to create the socioeconomic status quintiles among NICHE participant households based on a baseline census performed in March 2007. The analysis was conducted using an approach described elsewhere [25]. The aim was to identify statistically-significant differences in socioeconomic status quintiles among *upesi jiko* and three-stone stove users, as described under statistical considerations below. Village remoteness was coded locally by an enumeration supervisor who estimated distances to primary services (county councils, main markets, and health centers) and characterized types of roads or paths (i.e., paved or dirt). Generally, urban villages were located within 5 kilometers of services and accessed by paved roadways, remote villages were 6-10 kilometers from services along dirt roads, and very remote villages were >10 kilometers from services along roads that are inaccessible during rainy seasons.

Project data were analyzed in *SAS* version 9.2 (SAS Institute, Inc., Cary, NC). Chi-square tests were used to identify statistically-significant differences in stove location, perceived benefits, and equity of adoption among *upesi jiko* and three-stone stove users. Significance was defined at a 0.05 alpha level.

## Results

### Perceived benefits of cookstoves

In June 2008, nearly all (98%) of 1,250 baseline survey respondents (response rate = 83%) indicated that they desired smoke reductions in their homes. Most respondents reported health concerns related to three-stone stove use, including breathing smoke (80%), burns (37%), and respiratory infections (11%). Nearly all (>99%) respondents reported using three-stone stoves for cooking. Almost three-fourths (72%) of respondents cooked inside the home (*n* = 895). One-fourth (28%) reported cooking in a kitchen located in the same room where people lived or slept (Table 1). Firewood was used as a fuel source by 95% of respondents and used exclusively by 36%; other types of biomass, including crop waste (42%) and charcoal (32%), were also commonly reported. Non-biomass fuels were used infrequently (paraffin: 3%, liquid petroleum gas: <1%). Most (78%) respondents who reported firewood expenditures spent at least 100 KSh per week. Median daily times spent collecting firewood and cooking were 2 hours (range: 0–9 hours) and 5 hours (range: 0–8 hours), respectively.

**Table 1:** **Cooking locations, expenditures, and smoke exposure at baseline and follow-up, Nyanza Province, Kenya, July 2008–December 2009 **^**a**^

	**Baseline survey**		**Follow-up survey**		
	**Three-stone stove**		**Three-stone stove**	***Upesi jiko***	**P-value**^**b**^
	**(*n*** **= 1250)**		**(*n*** **= 202)**	**(*n*** **= 91)**	
	***N*(%)**		***N*(%)**	***N*(%)**	
**Cooking location**^**c**^					0.0002
Indoors, kitchen as separate room	210 (16.8)		38 (18.9)	20 (22.0)	
Indoors, same room where people live/sleep	351 (28.1)		68 (33.8)	15 (16.5)	
A separate building dedicated for cooking	613 (49.0)		74 (36.8)	54 (59.3)	
Outdoors/outside the house	425 (34.0)		21 (10.5)	2 (2.2)	
**Money spent on firewood (shillings/week)**^**d**^					0.004
1–99	110 (22.3)		40 (23.0)	34 (40.5)	
100–199	205 (41.6)		85 (48.9)	25 (29.8)	
200 or more	178 (36.1)		49 (28.2)	25 (29.8)	
**Total time spent collecting firewood (hours/day)**^**e**^					0.79
1 or less	315 (27.0)		77 (49.0)	29 (45.3)	
2	466 (39.9)		69 (44.0)	29 (45.3)	
3 or more	305 (26.1)		11 (7.0)	6 (9.4)	
**Total time spent cooking (hours/day)**					0.19
Less than three	28 (2.2)		–	–	
3–4	448 (35.8)		96 (48.5)	43 (50.0)	
5–6	675 (54.0)		90 (45.5)	42 (48.8)	
7 or more	99 (7.9)		12 (6.1)	1 (1.2)	
**Children <2 years of age present while cooking**^**f**^					0.27
Yes, always	333 (42.3)		29 (29.0%)	18 (41.9%)	
Yes, sometimes	264 (33.5)		53 (53.0%)	17 (39.5%)	
Never	191 (24.2)		18 (18.0%)	8 (18.6%)	
**Daily smoke exposure duration for children <2 years of age**					0.01
60 minutes or less	57 (10.0)		64 (78.0%)	21 (61.8%)	
60–120 minutes	245 (43.1)		18 (22.0%)	10 (29.4%)	
120 minutes or more	267 (46.9)		0	3 (8.8%)	
**Children 2 to <5 years of age present while cooking**^**f**^					0.27
Yes, always	375 (37.0)		31 (22.3%)	14 (35.0%)	
Yes, sometimes	517 (51.0)		88 (63.3%)	21 (52.5%)	
Never	121 (11.9)		20 (14.4%)	5 (12.5%)	
**Daily smoke exposure duration for children 2 to <5 years of age**					0.02
60 minutes or less	64 (7.2)		101 (84.2%)	21 (61.8%)	
60–120 minutes	439 (49.2)		17 (14.2%)	12 (41.4%)	
120 minutes or more	390 (43.7)		2 (1.7%)	1 (2.9%)	
**Children 2 to <5 years of age sleep in kitchen at night**					0.01
Yes, every night	617 (53.2)		48 (34.5%)	5 (12.8%)	
Yes, sometimes/rarely	48 (4.1)		11 (7.9%)	1 (2.6%)	
No, never	495 (42.7)		80 (57.6%)	33 (84.6%)	

^a^ Stove types are *upesi jiko* (ceramic-lined cookstoves) vs. three-stone stoves (traditional firepits with surrounding stones).

^b^ P-values are for *χ*^2^ tests of association between stove type and cooking locations, expenditures, and smoke exposure in follow-up survey.

^c^ Cooking locations sum to >100% in baseline survey because of multiple locations. Primary cooking locations analyzed for follow-up survey.

^d^ One hundred Kenyan shillings was approximately 1.25 U.S. dollars in 2008.

^e^ Eighty-three (7.1%) respondents reported spending no time collecting firewood in the baseline survey.

^f^ Among 788 (63.0%) households with children less than 2 years of age or 1013 (81.0%) households with children 2 to less than 5 years of age.

Three-quarters (76%) of households with children less than two years of age and most (88%) households with children two to less than 5 years of age reported that their infants or young children were always or sometimes present while cooking (Table 1). Median daily durations of smoke exposures were 2 hours each for children less than two years (range: 0–9 hours) and children two to less than 5 years of age (range: 0–8 hours). Half (53%) of children two to less than 5 years of age slept in the kitchen nightly.

In the 10 villages that participated in the pilot stage of the project, 91 (31%) respondents who used *upesi jiko* were compared with 202 (69%) respondents who participated in NICHE and had continued to use three-stone stoves during the follow-up survey in January and February of 2009 (Table 1). *Upesi jiko* were more often (59%) located in a separate building for cooking compared with three-stone stoves (37%), while cooking in the same room where people live or sleep was more common for users of three-stone stoves (34%) than *upesi jiko* (17%) (p = 0.0002). *Upesi jiko* users (40%) were more likely than three-stone stove users (20%) to indicate that they spent less than 100 KSh (~$1.25 USD) per week on firewood (p = 0.004), but both types of stove user often (>58%) spent at least 100 KSh weekly on firewood. Total time spent collecting firewood (p = 0.79) or cooking (p = 0.19) did not differ between *upesi jiko* and three-stone stove users.

During the follow-up survey, there were no significant differences in the presence of children less than two years of age in households using *upesi jiko* (48%) or three-stone stoves (49%) (p = 0.88) (data not shown). However, children two to less than 5 years of age were less common in households using *upesi jiko* (46%) compared with households using three-stone stoves (69%) (p = 0.0001). Unlike the baseline survey, infants’ and young children’s daily durations of smoke exposures did not typically exceed two hours in all households (Table 1). Fewer households using *upesi jiko* (13%) or three-stone stoves (35%) reported children two to less than 5 years of age sleeping in the kitchen every night when compared with the baseline survey (53%).

### Rate of cookstove adoption

From July 2008 through December 2009, SWAP vendors sold and installed 1,124 *upesi jiko* among 757 multi-family households in the 60 villages (Figure2); 60% of households installed a single *upesi jiko*, 38% installed two, and 2% installed three or four. In the initial 10 villages (i.e., the pilot stage), 193 (17%) *upesi jiko* were sold and installed. During the expansion stage, 922 (82%) *upesi jiko* were sold and installed in all 60 villages as the project incorporated the remaining 50 villages. Among 5,868 total households in the villages, overall adoption (i.e., the percentage of village households with at least one *upesi jiko* installed) was similar in the pilot (median: 8%) and expansion (median: 10%) stages, but varied substantially among the villages (range: 1–78%).

One to six of the 160 SWAP vendors resided in each of the 60 villages. Twenty-eight (31%) SWAP vendors were involved in a single *upesi jiko* transaction. Among SWAP vendors with multiple transactions, the median number of *upesi jiko* transactions was seven (interquartile range: 1–10). Median total costs of *upesi jiko* (including sale and installation) for households were 150 KSh (range: 50–500). The top vendor, who had 89 transactions, was active throughout the duration of the project and operated in 14 villages.

In the 60 villages’ households, the majority (76%) of *upesi jiko* installations occurred from August through October of 2009 (i.e., near the end of the expansion stage), which was after the second round of incentives and offers were widely established to accelerate *upesi jiko* transactions (Figure2). In September and October of 2009, >300 *upesi jiko* were installed each month. Overall, two-thirds (68%, *n* = 759) of transactions involved promotional offers or incentives for households, especially pricing incentives that partially subsidized the transaction (*n* = 645 transactions). During the pilot stage, most sales (*n* = 178 *upesi jiko*, 84%) followed the promotion in October 2008. Also, combining sales and installations to a single transaction reduced the median time from sale to installation from 6 days (IQR: 1–12 days) in the pilot stage to a median of one day (IQR: 0–3 days) in the expansion stage (p < 0.01).

Measures of household water treatment consistently demonstrated enhanced adoption of *upesi jiko* in households that had been treating their household water more frequently (i.e., integration). Specifically, adoption was more common in households where water treatment in the last 12 hours had been reported frequently (11%) or sporadically (10%) compared with households that had never (4%) reported treating their water during previous study visits (p = 0.0003). When treatment was confirmed by measuring chlorine residuals in stored water, *upesi jiko* adoption also was more common in households where chlorine had been detected frequently (17%) or sporadically (10%) compared with households where chlorine had never (5%) been detected (p = 0.004).

### Equity of cookstove adoption

Nearly half (49%) of *upesi jiko* were installed in households of women in the oldest quartile for age (>31 years, p < 0.0001) (Table 2). Installations of *upesi jiko* did not differ by education level of the female head of household (p = 0.98). Two-thirds (66%) of *upesi jiko* installations were concentrated among households with the two highest quintiles of socioeconomic status (i.e., least poor); this socioeconomic status distribution among *upesi jiko* differed significantly when compared to the socioeconomic status distribution among three-stone stove users (p < 0.0001). Relatively few (<10%) *upesi jiko* were installed in households of women in the youngest age quartile (<22 years) or among households in the poorest quintile. Most *upesi jiko* installations occurred in villages that were considered either remote (49%) or very remote (36%), which was consistent with the overall frequency of urban (19%), remote (44%), and very remote (37%) locations among the NICHE project villages.

**Table 2:** **Demographic and household socioeconomic characteristics by stove type, Nyanza Province, Kenya, July 2008–December 2009 **^**a**^

	***Upesi jiko***	**Three-stone stove**	
	***N*(%)**	***N*(%)**	**P-value**^**b**^
**Woman’s age (years)**^c^			<0.0001
< 22	6 (6.7)	312 (23.1)	
22–25	20 (20.2)	343 (25.4)	
26–31	20 (20.2)	364 (27.0)	
>31	44 (48.9)	330 (24.5)	
**Woman’s education**^c^			0.98
None or some primary school	44 (48.9)	660 (49.0)	
Completed primary school or more	46 (51.1)	687 (51.0)	
**Household socioeconomic status quintiles**^**d**^			<0.0001
0 (most poor)	12 (9.8)	353 (20.3)	
1	14 (11.4)	377 (21.6)	
2	16 (13.0)	309 (17.7)	
3	39 (31.7)	353 (20.3)	
4 (least poor)	42 (34.2)	351 (20.1)	

^a^ Stove types are *upesi jiko* (ceramic-lined cookstoves) vs. three-stone stoves (traditional firepits with surrounding stones). Household data on stove type were linked to demographic and socioeconomic data for 158 (20.9%) of 757 households with *upesi jiko* and a random sample of 1,782 households with three-stone stoves.

^b^ P-values are for *χ*^2^ tests of association between stove type and demographic and socioeconomic factors.

^c^ Primary caregiver of children in the household. Age and education categorized based on quartiles and the approximate median, respectively. Data were missing for 667 (88.1%) households with *upesi jiko* and 435 (24.4%) households with three-stone stoves.

^d^ A principal component analysis of household assets (e.g., household goods, building structure) was used to create quintiles of socioeconomic status (SES) among NICHE participant households. SES data were missing for 634 (83.8%) households with *upesi jiko* and 39 (2.2%) households with three-stone stoves.

## Discussion

Our evaluation demonstrated that a strategy of mobilizing local vendors, using pricing incentives and promotional offers, and integration with household water treatment accelerated adoption of locally-produced, ceramic cookstoves (*upesi jiko*) into a large number of rural Kenyan households. While there is an abundance of literature on improved cookstoves to mitigate household air pollution, this report offers a description of the specific processes and mechanisms for delivering cookstoves to households. Given that rates of cookstove adoption may be low and the factors that determine adoption are not yet well understood [26], similar evaluations are needed for other improved cookstove technologies in other settings. Furthermore, lessons from programs that have successfully delivered other types of interventions (e.g., insecticide-treated bed nets, micronutrient supplementation packets) may be extrapolated to cookstoves.

The strategy can be understood in terms of the theories of Rogers’s *Diffusion of Innovations*[27]. The networks of SWAP vendors accelerated the rate of adoption of the *upesi jiko*, both by acting as persuasive community peers, with roles as opinion leaders, and by providing specific health and product knowledge that was transferred during project trainings and amplified through interpersonal communication channels. These same SWAP vendors were motivated by the potential for modest but meaningful income with the sale and installation of *upesi jiko*. In fact, ~70% of SWAP vendors were actively involved in the *upesi jiko* market, as evidenced by multiple transactions (i.e., a median of seven transactions). Also, pricing incentives and the promotional offers likely accelerated the adoption rate by increasing the relative advantage of *upesi jiko* compared with traditional, three-stone stoves. As a result, a market that was attainable for many village members was established. Although these incentives and promotions were not necessarily sustainable because they require external funding, they were relatively inexpensive and probably indispensible; two-thirds of *upesi jiko* transactions involved incentives/promotions during the project. Importantly, incentives and promotions were timed to follow crop harvests, which often provide extra, seasonal income. Other evaluations should determine whether the effects of pricing incentives are attributable to a lower absolute price or periodic price reductions acting as a stimulus.

Integration of environmental health interventions to improve air and water quality capitalized on existing NICHE and SWAP resources and infrastructure, and created additional opportunities to reinforce health messages and promote adoption of the *upesi jiko*. For community members who had already adopted household-level water treatment, the *upesi jiko* may have been compatible with an idea that was previously-introduced by SWAP; namely, products introduced at the household level can improve child survival. Furthermore, the *upesi jiko* were compatible with needs reported by the community (e.g., desired reductions in smoke) and, in general, were likely perceived to have some beneficial attributes (especially savings in firewood expenditures). At the program level, integration of interventions has the potential to be more cost-effective and sustainable than separate, vertical efforts [28,29]. However, the effects of adding new products such as cookstoves to the SWAP project need to be monitored for a potentially negative impact on the distribution of other products. The finding that *upesi jiko* adoption was greater in households that had been treating water more frequently is consistent with the hypothesis that health messages and corresponding product interventions reinforced one another. It may also be true that households that accepted one intervention may have been more likely to accept other interventions because of other underlying factors, such as higher socioeconomic status or a greater degree of health education. Notably, our evaluation was not designed to determine the relative impact of each intervention while co-promoting household water treatment and *upesi jiko* stoves.

Our findings should be interpreted cautiously, as demographic and socioeconomic data were linked to a very small subset of all households that used *upesi jiko*. The project resulted in adoption of *upesi jiko* by numerous village members. Although the stoves were promoted without regard to demographics or socioeconomic status (i.e., village residents had equal access to the *upesi jiko*), most of the *upesi jiko* were acquired by households in the uppermost socioeconomic status quintiles and by households where older women reside. The implications of these findings are significant. For example, many of these households may have larger homes with more rooms or buildings, which would allow them to install *upesi jiko* in separate buildings (i.e., away from where children sleep) more frequently than households with lower socioeconomic status. Also, we did not have adequate data to determine the proportion of households with young children that were able to install an *upesi jiko*. Therefore, the overall success of the project so far has been diminished by the inequitable adoption of the cookstoves [30] and possibly by incompletely reaching the intended target population, young children, for reduction of exposures to household air pollution [31,32]. Although demographic and socioeconomic characteristics are important determinants, other research in Africa suggests that the attributes of the cookstoves themselves also determine whether cookstoves will be adopted at the household level [33]. Indeed, adoption of improved cookstoves is a complex process; even when new cookstove technologies are adopted, they may be used in addition to (rather than instead of) traditional alternatives [34]. A subsequent evaluation will determine whether the adoption of *upesi jiko* has had long-term success though the ongoing, appropriate use of the *upesi jiko*. Furthermore, it is understood that our evaluation of a multifaceted strategy, including local vendors, promotional incentives, and product integration, did not allow for separate assessments of the impacts of each of these components of the strategy. Specific limitations also may apply to each component of the evaluation. For example, water treatment was generally categorized with consistency when measured through self-reporting and chlorine residuals, but discrepancies were identified. The largest discrepancies were households with reporting that suggested sporadic treatment, but chlorine residuals indicated frequent treatment (37%) and households with reporting that suggested sporadic treatment, but chlorine residuals indicated no treatment (34%).

Although this project effectively incorporated these cookstoves into a large number of households of Luo ethnicity, additional applications will be needed to determine whether a strategy of local vendors, promotional incentives, and product integration can be applied equitably in other communities. Planning and design of these programs should include formal evaluations to determine the effectiveness of each component of the stove-adoption strategy. A considerable body of emerging research is determining whether reductions in fuel use, household air pollution, and the incidence of respiratory diseases result from the use of improved cookstoves. At the same time, integrated, scalable strategies for improving access to cookstoves in rural households are also needed to accomplish these reductions.

## Conclusions

We implemented a project to promote, sell, and install locally-produced, ceramic cookstoves in an impoverished area of rural western Kenya. Over 1,100 *upesi jiko* were adopted by households in 60 villages during an 18-month evaluation period. Following the evaluation, sales and installations of these cookstoves continued at an approximate rate of 25 units per month, reaching 2,014 cookstoves by the end of 2010. The project was accomplished through a combined strategy that included local vendors, promotional offers and pricing incentives, and product integration of *upesi jiko* with household water treatment products. Interventions to reduce exposure to household air pollution are critically important in regions such as this because the vast majority of households cook over open firepits with firewood or other biomass and because infants, young children, and women are frequently present during cooking.

## Competing interests

The authors declare that they have no competing interests. The findings and conclusions in this article are those of the authors and do not necessarily represent the views of the Centers for Disease Control and Prevention.

## Authors’ contributions

BJS, REQ, and ALC contributed to all aspects of the program design, implementation, analyses, and reporting. IS, JH, and RO coordinated fieldwork and data collection. IS, MKP, VW, and BN managed and analyzed study data. JL, BP, and AE provided technical guidance for program design and implementation. All authors contributed to the design and evaluation of the program and read and approved the final manuscript.

## Pre-publication history

The pre-publication history for this paper can be accessed here:

http://www.biomedcentral.com/1471-2458/12/359/prepub
